# Diagnostic evaluation of the relationship between impacted mandibular third molars and the mandibular canal in panoramic radiographs shared via WhatsApp

**DOI:** 10.1007/s11282-026-00938-2

**Published:** 2026-06-09

**Authors:** Jerusa Jobim Jardim, Heraldo Luis Dias da Silveira, Nádia Assein Arús, Priscila Fernanda da Silveira Tiescher, Deise Ponzoni, Mariana Boessio Vizzotto

**Affiliations:** Surgery and Orthopedics Department, Federal University of Rio Grande do Sul, Av. Ramiro Barcelos 2492, 5th Floor, Porto Alegre, 90035000 Brazil

**Keywords:** Smartphone, Mobile applications, Molars, third, Panoramic radiography

## Abstract

**Objective:**

To compare the diagnostic accuracy of computer and smartphone screens for assessing the radiographic proximity between impacted mandibular third molars and the mandibular canal.

**Methods:**

Four examiners evaluated 121 digital panoramic radiographs for the presence of proximity between third molars and the mandibular canal, as well as for specific radiographic signs indicative of this relationship. Assessments were performed on a computer screen and on their personal smartphone screens; for the latter, images were shared via WhatsApp. Diagnostic performance was analyzed using accuracy (Ac), sensitivity (Se), specificity (Sp), and positive and negative predictive values (PPV and NPV). Differences in the frequency of correct and incorrect responses between methods were tested using the McNemar test, as were the proportions of correct and incorrect classifications for each radiographic sign.

**Results:**

Examiners correctly classified true positives and true negatives in 86.5% of cases using the computer monitor and 85.1% using the smartphone. Comparable diagnostic performance was observed for computer monitor (Se = 0.975; Sp = 0.758; Ac = 0.865) and smartphone (Se = 0.942; Sp = 0.762; Ac = 0.851) assessments. No statistically significant differences were found between methods for any radiographic sign (*p* > 0.05).

**Conclusion:**

Smartphones demonstrated diagnostic accuracy comparable to that of computer screens for evaluating the proximity between impacted mandibular third molars and the mandibular canal on panoramic radiographs.

## Introduction

In dentistry, high diagnostic accuracy is essential, and obtaining a second opinion can be helpful for complex cases. Consequently, sharing radiographic images via instant messaging on smartphones is common among health professionals. Sarode et al. [1] found that 98% of dentists regularly used instant messaging apps to send clinical photographs and radiographs for second opinions. WhatsApp is one of the most widely used apps, allowing rapid message transmission across nearly all mobile operating systems [2].

Wani et al. [3] first evaluated WhatsApp for healthcare communication in plastic and reconstructive surgery, finding it effective, inexpensive, fast, and easy to use. Subsequent studies confirmed its effectiveness for medical information exchange and reliability in analyzing radiographic images for diagnostics [4–9]. The European General Data Protection Regulation [10] mandates specific rules to protect patients’ rights, but no global guidelines exist for WhatsApp’s use in clinical practice [11, 12].

Extraction of the mandibular third molar is a common oral surgery [13, 14]. Preoperative evaluation of the relationship between an impacted third molar and the mandibular canal is crucial. Panoramic radiography is the preferred imaging technique due to its availability, ease of use, low cost, and low radiation dose [15]. Despite some overlapping and distortion, it is recommended for evaluating the surgical site and identifying perioperative risks [13, 16, 17]. Specific high-risk signs include interruption of the radiopaque line of the mandibular canal wall, deviation or narrowing of the mandibular canal, and a radiolucent area over the molar roots, all indicating close anatomical proximity and a higher risk of inferior alveolar nerve injury [17–22].

The European Society of Radiology [23] notes that viewing radiographic images on mobile devices is not recommended for primary interpretation; it facilitates sharing for second opinions. The accuracy of diagnosing the relationship between the included mandibular third molars and the mandibular canal using WhatsApp remains unclear. No studies specifically address this issue. Given the growing use of mobile image sharing for better diagnoses, further research is warranted.

## Materials and methods

This cross-sectional observational study was conducted at the Diagnostic Imaging Laboratory of the School of Dentistry at the Federal University of Rio Grande do Sul (UFRGS). The study adhered to the guidelines outlined in Resolution 466/2012 of the National Health Council, which governs research involving human subjects. Prior to initiation, the study received approval from the Research Ethics Committee of UFRGS (CEP-UFRGS), under the substantiated CAAE No. 56710022.0.0000.5347.

### Case selection

A sample size of at least 112 mandibular third molars was calculated to test for a minimum difference of 0.15 points between the AUC (area under the curve) of the methods under analysis, with 80% power and 5% significance. The calculation utilized the ‘power.roc.test’ function from the pROC package in R. To ensure robustness and the largest required sample size, we assumed A1 = A2 = 0 (mean test scores in both positive and negative conditions equal), B1 = B2 = 1 (test scores in both conditions have the same variability), and RN = ra = 0.5 (equal prevalence of negative and positive conditions).

This study utilized 121 panoramic radiographs, each measuring 2864 × 1348 pixels with a resolution of 336 dpi. These images were obtained using a flat-panel digital sensor and a Carestream CS8100 dental X-ray machine from the image bank of the Digital Image Processing Laboratory (LAPID) at the UFRGS School of Dentistry. The study included panoramic radiographs with diagnostic quality that clearly showed mandibular third molars with complete rhizogenesis. The sample was divided into two groups. Only one mandibular third molar per radiograph was included. The first group consisted of 60 images of fully formed, impacted mandibular third molars whose root apices were in proximity to the mandibular canal. The second group comprised 61 images in which the molars showed no close anatomical relationship with the mandibular canal. The reference standard was established by consensus through the analysis of radiographs by three professors from the UFRGS School of Dentistry: two from Oral Radiology (MBV and NAA) and one from Oral and Maxillofacial Surgery and Traumatology (DP). Each case was evaluated for signs of direct contact between the third molar and the mandibular canal, and for specific predictive radiographic signs of proximity, including: (a) interruption of the canal wall’s radiopaque line, (b) radiolucent band or area over the third molar roots, (c) deflection of the canal around the root apices, and (d) narrowing of the roots.

### Image evaluation

All students enrolled in the Integrated Residency Program in Oral Health at the School of Dentistry, with an emphasis on Oral and Maxillofacial Surgery and Traumatology, were invited to participate in the study. The examiner group consisted of six residents with experience in interpreting panoramic radiographs. The Coordination of the Integrated Residency Program in Oral Health at the School of Dentistry was informed of the residents’ voluntary involvement in this research. Students who agreed to participate were instructed to read and sign the informed consent form (ICF). A total of 121 radiographs comprised the sample, and these were randomized for each of the two analysis stages using the Research Randomizer application (https://www.randomizer.org).

In M1, the radiographs and their corresponding questions were compiled in a Google Form, with each image followed by two sequential case-related questions. Examiners viewed and answered the questions on a computer monitor and submitted the completed form electronically.

In M2, a separate Google Form containing only the questions was emailed to the examiners. The radiographs, identified by case and tooth numbers, were shared via WhatsApp in JPG format and were accessed and analyzed on smartphone screens. The examiners subsequently completed the questions and submitted the form electronically.

Both methods used panoramic radiographs with a resolution of 2864 × 1348 pixels, 336 DPI, and a file size of approximately 1.16 megabytes (1,219,622 bytes), shared in JPG format. In each visualization method, the examiners answered two questions for every case analyzed. The first asked them to determine whether there was direct contact between the included molar and the mandibular canal, using a five-point Likert scale: YES, certainly; YES, probably; I DON’T KNOW; NO, probably not; and NO, definitely not. For the second question, if the examiner responded “yes,” they were instructed to select one or more predictive radiographic signs of proximity.

### Statistical analysis

All analyses were conducted using the R language [24]. Accuracy, sensitivity, specificity, positive predictive value (PPV), and negative predictive value (NPV) were calculated to compare evaluations with the gold standard. McNemar’s χ² test was used to compare discordance between two dichotomous variables, particularly to compare sensitivity and specificity between diagnostic methods in paired data. Sensitivities were compared based on evaluation proportions of each method in positive diagnosis cases, while specificities were compared among negative diagnosis cases using McNemar’s test [25]. Cohen’s Kappa coefficient measured agreement between the evaluations made by the same evaluator on two different occasions. Agreement levels were classified as low (0 < K < 0.20), reasonable (0.21 < K < 0.40), moderate (0.41 < K < 0.60), substantial (0.61 < K < 0.80), and perfect (0.81 < K < 1.0), with K < 0 indicating no agreement [26]. A significance level of α = 0.05 was applied to all analyses, and p-values at or above this threshold were interpreted as evidence to retain the null hypothesis.

## Results

Table 1 presents the intra-rater agreement analysis using Cohen’s Kappa statistics. Evaluators completed the questionnaire using method 1 twice at different times, and agreement between their responses was assessed. The original 5-level response scale collapsed into three categories: Yes (definitely yes and probably yes), No (definitely not and probably no), and don’t know. Kappa (K) values ranged from 0.54 to 0.88. Analyses of the four highest agreement values were used in the statistical analysis.

Table 2 displays precision measures of evaluations compared to the gold standard for both methods. Accuracy rates were 86.5% using computer screen and 85.1% for smartphone (using WhatsApp). Computer visualization outperformed smartphones in accuracy, sensitivity, PPV, and NPV, while smartphones showed higher specificity. Despite this, most metric values were similar between the two methods. Both methods demonstrated high accuracy, sensitivity, specificity, PPV, and NPV, ranging from 0.758 to 0.975, indicating good evaluation accuracy compared to the gold standard. Table 3 presents the frequencies of “yes” and “no” responses for each method and compares the proportions using the McNemar test. This statistical approach allows for a comparison of sensitivity and specificity values shown in Table 2.

The McNemar test indicates that the difference in sensitivities was significant (*p* = 0.0133), while the difference in specificities was not (*p* = 1). This means that computer visualization was significantly more sensitive than smartphone using WhatsApp. Table 3 also displays the overall frequencies of “yes” and “no” responses. The McNemar test result was not significant (*p* > 0.05), suggesting no significant difference in the proportion of responses between Method 1 and Method 2. Thus, the change in response proportions between the methods was not statistically significant.

**Fig. 1:**
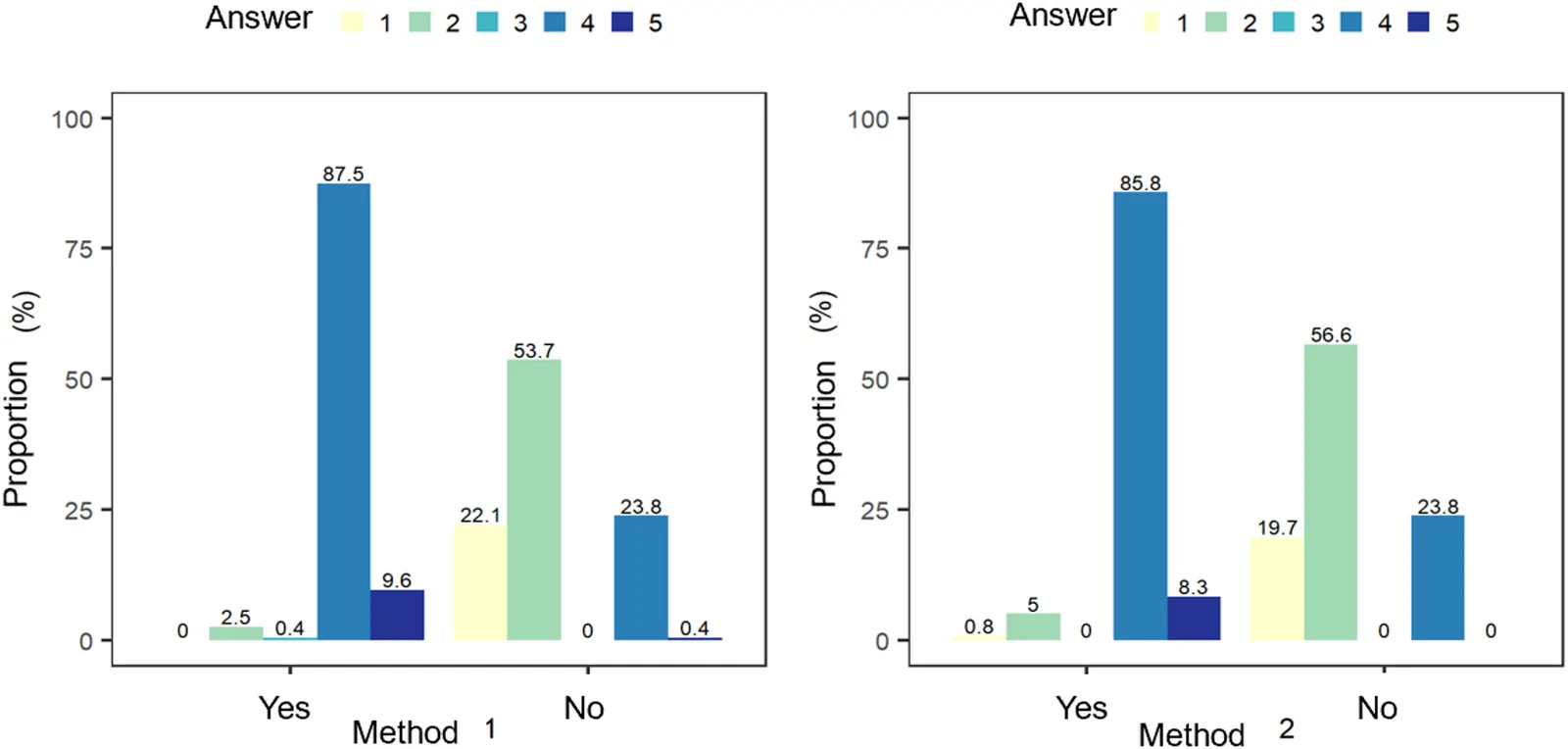
Distribution of evaluators’ responses in relation to the gold standard by method on a scale from 1 (No, there is definitely no direct contact) to 5 (Yes, there is definitely direct contact)

Figure 1 illustrates the distribution of evaluators’ responses on the Likert scale relative to the gold standard for each method, allowing comparison of response proportions. For the gold standard “NO,” most responses were “NO, probably no direct contact” in both computer screen (53.7%) and smartphone using WhatsApp (56.6%). For the gold standard “YES,” the predominant response was “YES, there is probably direct contact,” with proportions of 87.5% and 85.8% for computer screen and smartphone using WhatsApp, respectively. These results suggest a tendency for evaluators to favor responses indicating probability over certainty.

The study proceeded with the evaluation of four radiographic signs identified by the evaluators: (1) “Radiolucent area over the roots of the third molar,” (2) " Narrowing of the roots of the third molar,” (3) “Deviation of the mandibular canal,” and (4) “Discontinuity of the cortical bone of the mandibular canal.” Table 4 presents the frequencies for each radiographic sign across the two methods, along with the total number of cases and the significance values from McNemar’s test. Overall, these results suggest no statistically significant difference between the proportions of correct and incorrect responses for any radiographic sign between the two methods.

**Table 1:** Analysis of intra-rater agreement in method 1 at two different times according to Cohen’s Kappa statistic (K) for each evaluator

Evaluator	K	CI 95%		*p*	Category of concordance [26]
1	0.55	0.41	0.68	< 0.001	Moderate
2	0.88	0.80	0.97	< 0.001	Perfect
3	0.87	0.78	0.96	< 0.001	Perfect
4	0.69	0.54	0.85	< 0.001	Substancial
5	0.54	0.39	0.69	< 0.001	Moderate
6	0.72	0.59	0.84	< 0.001	Substancial

**Table 2:** Measures of accuracy of the evaluations in comparison with the gold standard, considering all evaluators in the two methods used in the study

Measurement	Method 1	Method 2
Accuracy	0.865	0.851
CI 95%	(0.832, 0.895)	(0.816, 0.882)
Sensitivity	0.975	0.942
Specificity	0.758	0.762
Positive predictive value	0.798	0.796
Negative predictive value	0.969	0.930
N	483	484

**Table 3:** Comparison of frequencies of “yes” and “no” responses between the two methods in reference to the gold standard and significance of the McNemar test

Gold Standard	Methods		Method 2			X² McNemar (*p*)
			No	Yes	Total	
Yes	Method 1	No	6	0	6	0.0133
		Yes	8	225	233	
		Total	14	225	239	
No	Method 1	No	173	12	185	1
		Yes	13	46	59	
		Total	186	58	244	
Both	Method 1	No	179	12	191	0.1637
		Yes	21	271	292	
		Total	200	283	483	

**Table 4:** Tables of frequencies of hits and misses between the two methods for each radiographic signal and significance of the comparison according to McNemar’s test

Radiographic sign	Method 1	Method 2			Total		Teste de McNemar (*p*)	
		Correct	Incorrect					
1) Radiolucent area	Correct	102	5		107		0.7237	
	Incorrect	3	2		5			
	Total	105	7		112			
2) Narrowing of the roots	Correct	30	7		37		0.6276	
	Incorrect	10	29		39			
	Total	40	36		76			
3) Deviation of the canal	Correct	16	4		20		1	
	Incorrect	5	15		20			
	Total	21	19		40			
4) Discontinuity of the cortical bone	Correct	31	13		44		0.3827	
	Incorrect	8	24		32			
	Total	39	37		76			

## Discussion

Smartphones are the most widely used devices for sharing and viewing images [27]. They have high-speed processors, large memory, high-resolution screens, fast internet, and free access to apps like WhatsApp. These features facilitate quick consultations for second opinions, providing fast and effective access to specialized knowledge. In Oral Radiology, sharing radiographs instantly can speed up diagnosis and treatment [28]. However, the reliability of radiographic images shared via WhatsApp is uncertain, as smartphone screens may not be ideal for viewing. This study assessed smartphone use for radiographic diagnosis and found an 85% accuracy rate in evaluating the mandibular third molar and mandibular canal relationship in panoramic images shared via WhatsApp. These findings are consistent with other studies on diagnostic accuracy using smartphone screens [27, 29–32].

Recent research indicates that the file format of periapical radiographs does not affect diagnostic accuracy for external and internal root resorption and vertical root fractures [33, 34]. In this study, the same file format was used for both methods. Madi et al. [30, 31] demonstrated WhatsApp’s excellent reliability for diagnosing jaw fractures and pathological conditions, with 99% accuracy and agreement (k) above 0.9. Comparing the two methods, computer screens were slightly superior overall. Both methods had high sensitivity values, indicating that panoramic radiographs are more reliable for confirming proximity between analyzed structures than excluding it, regardless of the screen used. Despite the limitations of two-dimensional examinations, computer screens had significantly higher sensitivity than smartphones, suggesting computer screens offer better conditions for identifying proximity between roots and the mandibular canal. Evaluators in this study identified multiple markers, contributing to high sensitivity values. According to Atieh [17], finding two or more radiographic markers improves sensitivity in predicting true proximity relationships.

This study identified radiographic markers predicting the proximity between the mandibular third molar and the mandibular canal. Evaluators most frequently noted the radiolucent area on the roots in both methods, consistent with previous findings [18]. Delamare et al. [35] found direct root-canal contact in 85% of cases with this marker. The radiolucent area often indicates a lingual position of the mandibular canal, associated with a higher risk of inferior alveolar nerve injury [16, 36] and higher incidence of post-extraction sensorineural deficits [14]. Comparing the proportions of correct answers between the two methods showed no statistically significant difference for any radiographic sign, suggesting equal difficulty in identifying markers across methods. Method 1’s greater sensitivity and frequent identification of multiple signs per case imply that identifying multiple markers is more crucial than the type of marker itself, aligning with previous research [17–22].

This study had limitations, including the lack of prior evaluator training and absence of strict ambient light control, to mimic clinical conditions. Computer monitors were uniform, and smartphones had standardized specifications to ensure diagnostic compatibility.

A major concern with sharing radiographs via WhatsApp is the potential exposure of sensitive patient information. Despite end-to-end encryption, WhatsApp lacks specific features for protecting medical data. Without global guidelines for such applications in healthcare, professionals must ensure data protection. The increasing use of WhatsApp in medicine highlights the need for regulation and monitoring of sensitive data sharing by competent authorities.

The diagnostic accuracy of panoramic radiographs shared via WhatsApp in smartphones for evaluating the relationship between impacted mandibular third molars and the mandibular canal was similar to that of computer screens. These findings align with current literature, highlighting smartphones as a reliable tool for dentists seeking cases discussion and second opinions.

## References

[1] Sarode SC, et al. Usage analysis of WhatsApp for dentistry-related purposes among general dental practitioners. J Contemp Dent Pract. 2018;19(10):1267–72. 30498184

[2] Giansanti D. WhatsApp in mHealth: an overview on the potentialities and the opportunities in medical imaging. mHealth. 2020;6:19. 10.21037/mhealth.2019.11.01. PMC7136653 32270011

[3] Wani SA, Rabah SM, Alfadil S, et al. Efficacy of communication amongst staff members at plastic and reconstructive surgery section using smartphone and mobile WhatsApp. Indian J Plast Surg. 2013;46(3):502–5. 10.4103/0970-0358.121990. PMC3897093 24459338

[4] Giordano V, Koch HA, Mendes CH, Bergamin A, de Souza FS, do Amaral NP. WhatsApp Messenger is useful and reproducible in the assessment of tibial plateau fractures: inter- and intra-observer agreement study. Int J Med Inform. 2015;84(2):141–8. 10.1016/j.ijmedinf.2014.11.002. 25468642

[5] Gross I, Langer Y, Pasternak Y, Abu Ahmad W, Eventov-Friedman S, Koplewitz BZ. Questionnaire-based study showed that neonatal chest radiographs could be reliably interpreted using the WhatsApp messaging application. Acta Paediatr. 2019;108(1):94–100. 10.1111/apa.14444. 29889988

[6] Kapıcıoğlu M, Erden T, Ağır M, Küçükdurmaz F. The reliability of use of WhatsApp in type 1 and type 2 pediatric supracondylar fractures. Eklem Hastalik Cerrahisi. 2019;30(2):149–54. 10.5606/ehc.2019.66166. 31291864

[7] Stahl I, Katsman A, Zaidman M, Keshet D, Sigal A, Eidelman M. Reliability of smartphone-based instant messaging application for diagnosis, classification, and decision-making in pediatric orthopedic trauma. Pediatr Emerg Care. 2019;35(6):403–6. 10.1097/PEC.0000000000001211. 28697157

[8] Ntja U, Janse van Rensburg J, Joubert G. Diagnostic accuracy and reliability of smartphone captured radiologic images communicated via WhatsApp®. Afr J Emerg Med. 2022;12(1):67–70. 10.1016/j.afjem.2021.11.001. PMC8761606 35070657

[9] Khoo SS, Guruseelan N, Gunasagaran J, Shamsul SA, Razak I, Ahmad TS. Reliability of WhatsApp images of plain radiographs to diagnose fractures of the hand and wrist. Journal of Hand Surgery (European Volume). 2023;48(5):419–25. 10.1177/17531934231151202. 36760195

[10] European Society of Radiology (ESR). The new EU General Data Protection Regulation: what the radiologist should know. Insights Imaging. 2017;8(3):295–9. 10.1007/s13244-017-0552-7. PMC5438318 28439718

[11] Mars M, Morris C, Scott RE. WhatsApp guidelines - what guidelines? A literature review. J Telemed Telecare. 2019;25(9):524–9. 10.1177/1357633X19873233. 31631763

[12] John B, McCreary C, Roberts A. Smartphone technology for communications between clinicians - a scoping review. J Dent. 2023;122:104112. 10.1016/j.jdent.2022.104112. 35413411

[13] Al-Haj Husain A, Stadlinger B, Winklhofer S, Bosshard FA, Schmidt V, Valdec S. Imaging in third molar surgery: a clinical update. J Clin Med. 2023;12(24):7688. 10.3390/jcm12247688. PMC10744030 38137758

[14] Rieder M, Remschmidt B, Schrempf V, Schwaiger M, Jakse N, Kirnbauer B. Neurosensory deficits of the mandibular nerve following extraction of impacted lower third molars-a retrospective study. J Clin Med. 2023;12(24):7661. 10.3390/jcm12247661. PMC10743649 38137730

[15] Leung YY, Cheung LK. Risk factors of neurosensory deficits in lower third molar surgery: an literature review of prospective studies. Int J Oral Maxillofac Surg. 2011;40(1):1–10. 10.1016/j.ijom.2010.09.005. 21035310

[16] Ghaeminia H, Meijer GJ, Soehardi A, Borstlap WA, Mulder J, Bergé SJ. Position of the impacted third molar in relation to the mandibular canal. Diagnostic accuracy of cone beam computed tomography compared with panoramic radiography. Int J Oral Maxillofac Surg. 2009;38(9):964–71. 10.1016/j.ijom.2009.06.007. 19640685

[17] Atieh MA. Diagnostic accuracy of panoramic radiography in determining relationship between inferior alveolar nerve and mandibular third molar. J Oral Maxillofac Surg. 2010;68(1):74–82. 10.1016/j.joms.2009.04.074. 20006158

[18] Rood JP, Shehab BA. The radiological prediction of inferior alveolar nerve injury during third molar surgery. Br J Oral Maxillofac Surg. 1990;28(1):20–5. 10.1016/0266-4356(90)90005-6. 2322523

[19] Umar G, Bryant C, Obisesan O, Rood JP. Correlation of the radiological predictive factors of inferior alveolar nerve injury with cone beam computed tomography findings. Oral Surg. 2010;3:72–82. 10.1111/j.1752-248X.2010.01088.x.

[20] Su N, van Wijk A, Berkhout E, et al. Predictive value of panoramic radiography for injury of inferior alveolar nerve after mandibular third molar surgery. J Oral Maxillofac Surg. 2017;75(4):663–79. 10.1016/j.joms.2016.12.013. 28041843

[21] Janovics K, Soós B, Tóth Á, Szalma J. Is it possible to filter third molar cases with panoramic radiography in which roots surround the inferior alveolar canal? A comparison using cone-beam computed tomography. J Craniomaxillofac Surg. 2021;49(10):971–9. 10.1016/j.jcms.2021.05.003. 34090736

[22] Bhardwaj P, Bhardwaj Y, Ram R, Parmar M, Ghezta N, Sinha A. Radiographic factors associated with inferior alveolar nerve exposure during mandibular third molar surgery and their influence on neurosensory deficit: a prospective study. J Oral Biol Craniofac Res. 2024;14(4):351–2. 10.1016/j.jobcr.2022.08.025. PMC9508473 36164406

[23] European Society of Radiology (ESR). ESR paper on the proper use of mobile devices in radiology. Insights Imaging. 2018;9(2):247–51. 10.1007/s13244-017-0589-7. PMC5893485 29569214

[24] R Core Team. R: A Language and Environment for Statistical Computing. Vienna, Austria: R Foundation for Statistical Computing; 2024.

[25] Trajman A, Luiz RR. McNemar χ2 test revisited: comparing sensitivity and specificity of diagnostic examinations. Scand J Clin Lab Invest. 2008;68(1):77–80. 10.1080/00365510701666031 18224558

[26] Landis JR, Koch GG. The measurement of observer agreement for categorical data. Biometrics. 1977;33(1):159–74. 843571

[27] Giacomini GO, Antonioli C, Tibúrcio-Machado CS, Fontana MP, Liedke GS. The use of smartphones in radiographic diagnosis: accuracy on the detection of marginal gaps. Clin Oral Investig. 2019;23(4):1993–6. 10.1007/s00784-019-02848-6. 30783793

[28] Madlum DV, Gaêta-Araujo H, Brasil DM, Lima CAS, Oliveira ML, Haiter-Neto F. Influence of the file format and transmission app on the radiographic diagnosis of caries lesions. Oral Surg Oral Med Oral Pathol Oral Radiol. 2021;132(4):448–55. 10.1016/j.oooo.2020.11.013. 33386287

[29] Cruz AD, Castro MC, Aguiar MF, Guimarães LS, Gomes CC. Impact of room lighting and image display device in the radiographic appearances of the endodontic treatments. Dentomaxillofac Radiol. 2018;47(5):20170372. 10.1259/dmfr.20170372. PMC6196042 29493288

[30] Madi M, Kumar M, Pentapati KC, Vineetha R, Chhaparwal Y. Email and instant messaging applications as platforms for remote oral radiology consultation in maxillofacial fractures. Oral Surg Oral Med Oral Pathol Oral Radiol. 2021;131(2):241–6. 10.1016/j.oooo.2020.07.007. 32768371

[31] Madi M, Kumar M, Pentapati KC, Vineetha R. Smart-phone based telemedicine: instant messaging application as a platform for radiographic interpretations of jaw pathologies. J Oral Biol Craniofac Res. 2021;11(3):368–72. 10.1016/j.jobcr.2021.04.003. PMC8093935 33996431

[32] Melo CA, Santos MAL, Menezes LS, Santana FS, Melo MFB, Takeshita WM. Use of smartphones and tablets in radiographic evaluation: accuracy of caries detection on bitewing radiographs. Gen Dent. 2021;69(4):28–34. PMID: 34185665. 34185665

[33] Miranda-Viana M, Madlum DV, Oliveira-Santos N, Gaêta-Araujo H, Haiter-Neto F, Oliveira ML. Influence of the image file format of digital periapical radiographs on the diagnosis of external and internal root resorptions. Clin Oral Investig. 2021;25(8):4941–8. 10.1007/s00784-021-03803-0. 33495894

[34] Miranda-Viana M, Fontenele RC, Farias Gomes A, et al. Digital file format does not influence the radiographic diagnosis of vertical root fracture. Oral Radiol. 2022;38(4):452–8. 10.1007/s11282-021-00573-z. PMC8501329 34626307

[35] Delamare EL, Liedke GS, Mariana Boessio Vizzotto MB, et al. Topographic relationship of impacted third molars and mandibular canal: correlation of panoramic radiograph signs and CBCT images. Braz J Oral Sci. 2012;11(3):411–5. 10.20396/bjos.v11i3.8641395.

[36] Tantanapornkul W, Okouchi K, Fujiwara Y, et al. A comparative study of cone-beam computed tomography and conventional panoramic radiography in assessing the topographic relationship between the mandibular canal and impacted third molars. Oral Surg Oral Med Oral Pathol Oral Radiol Endod. 2007;103(2):253–9. 10.1016/j.tripleo.2006.06.060. 17234544

